# Gene and miRNA expression changes in squamous cell carcinoma of larynx and hypopharynx

**DOI:** 10.18632/genesandcancer.69

**Published:** 2015-07

**Authors:** Jayalakshmi Nair, Prachi Jain, Udita Chandola, Vinayak Palve, N R. Harsha Vardhan, Ram Bhupal Reddy, Vikram D. Kekatpure, Amritha Suresh, Moni Abraham Kuriakose, Binay Panda

**Affiliations:** ^1^ Ganit Labs, Bio-IT Centre, Institute of Bioinformatics and Applied Biotechnology, Biotech Park, Electronic City, Bangalore, India; ^2^ Mazumdar Shaw Centre for Translational Research, Mazumdar Shaw Medical Centre, Narayana Hrudayalaya, Bangalore, India; ^3^ Strand Life Sciences, Bellary Road, Hebbal, Bangalore, India

**Keywords:** RNA-seq, squamous cell carcinoma, larynx, hypopharynx, gene expression, miRNA and promoter hypermethylation

## Abstract

Laryngo-pharyngeal squamous cell carcinomas are one of the most common head and neck cancers. Despite the presence of a large body of information, molecular biomarkers are not currently used in the diagnosis, treatment and management of patients for this group of cancer. Here, we have profiled expression of genes and microRNAs of larynx and hypopharynx tumors using high-throughput sequencing experiments. We found that matrix metalloproteinases along with SCEL, CRNN, KRT4, SPINK5, and TGM3 among others have significantly altered expression in these tumors. Alongside gene expression, the microRNAs hsa-miR-139, hsa-miR-203 and the hsa-miR-424/503 cluster have aberrant expression in these cancers. Using target genes for these microRNAs, we found the involvement of pathways linked to cell cycle, p53 signaling, and viral carcinogenesis significant (*P*-values 10^−13^, 10^−9^ and 10^−7^ respectively). Finally, using an ensemble machine-learning tool, we discovered a unique 8-gene signature for this group of cancers that differentiates the group from the other tumor subsites of head and neck region. We investigated the role of promoter methylation in one of these genes, WIF1, and found no correlation between DNA methylation and down-regulation of WIF1. We validated our findings of gene expression, 8-gene signature and promoter methylation using q-PCR, data from TCGA and q-MSP respectively.

Data presented in this manuscript has been submitted to the NCBI Geo database with the accession number GSE67994.

## INTRODUCTION

Head and neck squamous cell carcinomas are a diverse group of tumors that originate from anatomically different locations, including nasal cavity, sinuses, lips, mouth, salivary glands, and throat. Cancers of the upper aero-digestive tracts (oral cavity, pharynx and larynx) are the sixth most common cancer worldwide [1]. In addition to tobacco, lifestyle factors, dietary deficiencies, gastroesophageal reflux and infection with human papilloma virus (HPV) are other reported risk factors for larynx and hypopharynx cancers [2-5].

Large-scale molecular characterizations of HNSCC have been performed in recent years for different subsites [6-10]. Despite this, results on gene expression and microRNA studies in tumors, especially for hypopharynx tumors, are very scarce. Earlier expression studies, in larynx and hypopharynx cancers, using immunohistochemistry (IHC), quantitative-PCR (q-PCR) and cDNA microarray linked genes to processes like cell adhesion, cell proliferation, differentiation, migration, apoptosis, transcriptional regulation and/or angiogenesis [11-14]. Additionally, overexpression of MDM2 and ERB2 were described as predictors of loco-regional failure of chemoradiation in larynx carcinoma [15].

In this study, we present data from whole transcriptome analyses using next-generation sequencing experiments to profile gene and miRNA expression landscape in larynx and hypopharynx carcinoma samples. We divided our study into two sets of patients, one where we discovered the gene expression signature (discovery set, *N*=13) and the other where we validated our results (validation set, *N*=18). We used computational pipeline with sequencing data from the discovery set to identify the significantly over- and under-expressed genes in SCCL and SCCHP. Our study identified matrix metalloproteinases, KRT4, SPINK5 and TGM3 among others as the main genes with altered expression in the tumors studied. Among the micro RNAs, miRNA-139, miRNA-203 and miRNA-424/503 cluster had altered expression in larynx and hypopharynx carcinomas. We found the target genes for these miRNAs to be linked to processes like cell cycle, p53 signaling and viral carcinogenesis in larynx and hypopharynx carcinoma. Additionally, we inferred a unique 8-gene signature using a machine-learning algorithm as a potential diagnostic marker set for this group of cancer, which was further validated using data from the TCGA samples.

## RESULTS AND DISCUSSION

We sequenced whole transcriptomes of larynx and hypopharynx tumors using next generation sequencing technology and identified significantly and unique differentially expressed genes in those tumors. Out of the 13 tumors that we sequenced, all except two were primary tumors. We used sequencing-by-ligation chemistry to produce paired-end color-space 75×35bp reads for all tumor and matched normal samples. Details of the read QC and mapping statistics (total number of mapped reads, reads mapped to exonic, intronic and intergenic regions) are provided in Supplementary Table 3. Across all samples for the whole transcriptome study, between 5-30% of total reads were filtered by mapping to tRNA, rRNA, adaptor and repeat sequences, 9-18% of total reads did not map to the reference sequence due primarily to the presence of low quality of reads while 40-75% reads got mapped to the reference genome (Supplementary Table 3). We used the QC-filtered reads further to find gene fusion variants, analyze expression changes, and perform further statistical analysis using random forest.

### Gene fusions

**Figure 1:**
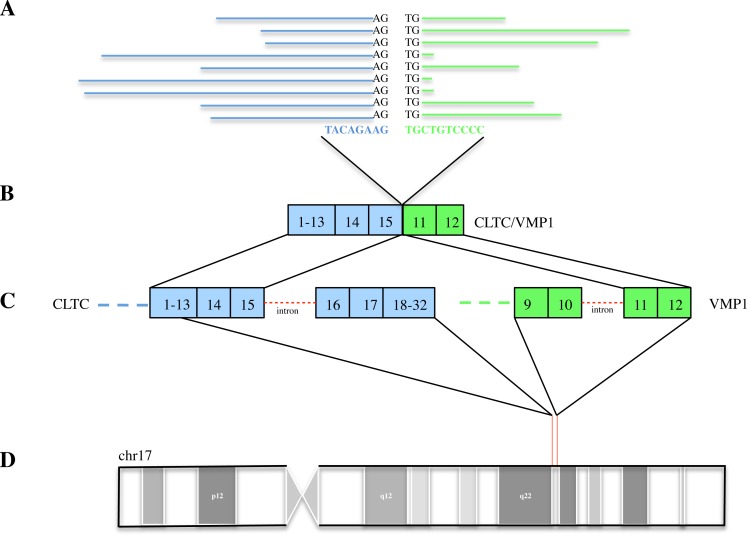
CLTC-CMP1 fusion in larynx and hypopharynx tumors **A.** Reads mapping the junction of CLTC(blue) and VMP1 genes (green). **B.** Junction of CLTC and VMP1 gene. **C.** Exon structures of CLTC and VMP1 genes. **D.** Chromosome 17 map with the location of CLTC-VMP1 gene.

We used the splice-finder module in Lifescope to detect gene fusion variants with a Junction Confidence Value (JCV) that aids in the detection of false positives. A number of gene fusions detected involved small nuclear/RNA genes (Supplementary Table 4). In one sample (a6f41n), we detected fusion between *CLTC-VMP1* genes, supported by 16 reads perfectly mapping across the breakpoint (Figure 1A). The observed CLTC-VMP1 fusion transcript was a result of fusions between the first 15 exons of CLTC and the last 2 exons VMP1 gene (Figure 1). *CLTC* gene has been previously implicated in gene fusion events in various leukemias, renal cell carcinoma, breast cancer and lung cancer [16-20]. VMP1 gene is an autophagy-related protein and the VMP1-dependent autophagy is shown to promote cell death in pancreatic cells [21]. Additionally, VMP1 is shown to be involved in drug sensitivity towards chemotherapeutic agents in pancreatic cell lines [22]. It is not known whether VMP1 has a similar effect in mediating chemotherapy-dependent apoptosis in larynx and hypopharynx cancers. Inaki et al (2011) previously reported recurrent expression of a fusion transcript involving *VMP1* (*RPS6KB1–VMP1*) in ~30% of breast cancers and the potential association of this expression with poor prognostic outcome in breast cancer patients [23]. In addition to *CLTC-VMP1* fusion, we also found an intra-chromosomal fusion event (JCV=68.52) involving CTBS-GNG5 genes in a tumor sample (Supplementary Table 4). The same fusion is reported previously in multiple cancer cell lines and primary tissues [24] but its functional consequences are currently not understood.

### Differentially expressed genes and experimental validation of genes in additional tumors

**Figure 2:**
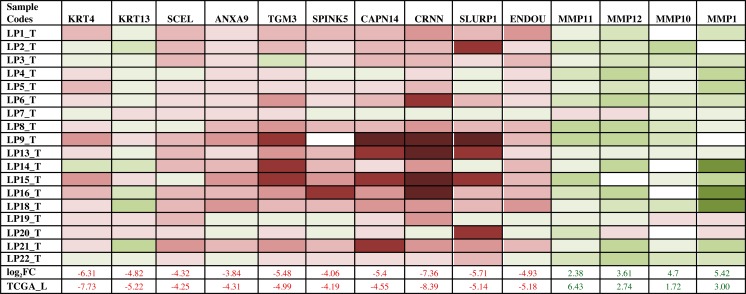
Quantitative PCR (qPCR) validation of gene expression changes, Red: down-regulation, Green: Up-regulation, shades of color is representative of the extent of expression changes in tumor samples compared to the matched normal tissues after normalization with internal beta-actin gene Log fold change (Log_2_FC) for the tumor samples (T) followed by validation from the larynx tumors from TCGA (TCGA_L) are also given in the last two columns.

We performed significance testing of the tumor vs normal FC values to find differentially expressed genes in the larynx and hypopharynx samples, selecting for those with a corrected *p*-value (padj) of at most 0.05 in either pooled or paired interpretations. A list of 47 significantly differentially expressed genes that passed the statistical test (padj≤0.05) and genes that are reported to play biological importance is given in Table 2. A complete list of 296 genes that were significantly and differentially expressed in tumors in either pooled or individual interpretations is provided in Supplementary Table 5. Most genes that met the criteria were down-regulated in tumor. Interestingly, most genes found to be significantly and differentially expressed in this study have also been implicated in SCCs of esophagus or oral cavity. This points to similar pathways being perturbed in cancers of the head and neck region. The gene WIF1 is down regulated in prostate, breast, lung, and bladder cancer [25] and probably indicates a higher risk of progression of disease with possible mechanisms through WIF1 promoter hypermethylation and loss of heterozygosity in salivary gland carcinoma [26]. In addition to WIF1, TGM3 is down-regulated in the larynx and pharyngeal samples. Previous results have shown that TGM3 is silenced by promoter hypermethylation in the cancers of head and neck[27]. Based on the results, we focused on 14 biologically important genes known to be cancer-associated for validation in additional tumors. We designed qPCR primers (Supplementary Table 2) and measured the expression profiles of these genes in a validation set with 18 tumors. The overall changes in gene expression among these samples correlated well with the sequencing data analysis results (Figure 2). Many of the genes from our list (MMPs, CRNN, KRT4, SPINK5, TGM3) are down regulated in other cancers, including in studies in HNSCC as shown previously [27-30]. Expression of many of these genes found in this study is already reported to change in different subsites of HNSCC [27, 30-33], for example, matrix metalloproteinases (MMPs), CRNN, KRT4, SPINK5, TGM3. In addition to the known genes, our study identified novel expression candidates in SCEL, ANXA9, CAPN14, SLURP1 and some MMPs. Genes like KRT4, SPINK5 and TGM3 are genes known to be frequently down-regulated in HNSCC [34]. These 3 genes are involved in epithelial/skin differentiation, structural and immune response pathways, similar to CRNN. KRT4 is co-expressed with KRT13 (also down-regulated in tumor samples in this study) in differentiated layers of the mucosal and esophageal epithelia [27-29]. Taken together, our results extend previously published information and finds additional novel expression markers in larynx and hypopharynx cancers.

### microRNA expression

We performed pooled analysis on miRNA differential expression analysis to find significant changes in their expression in tumor samples compared with the corresponding normal samples (Supplementary Table 5). We found four miRNAs with significant altered expression levels in the larynx and hypopharynx tumors sequenced. They are hsa-miR-139, hsa-miR-203, hsa-miR-424 and hsa-miR-503. Out of these, microRNAs miR-424 and miR-503 belong to the same cluster (miR-424/503). The miRNA hsa-miR-139 and hsa-miR-203 are down regulated and hsa-mir-424 and hsa-miR-503 are up regulated in tumor samples (Supplementary Table 5). Previous studies have established the role of miRNA expression in different cancers including in HNSCC [35-43]. Despite this diverse body of information, the expression changes in larynx and hypopharynx carcinoma and their exact role in carcinogenesis have not been universally accepted. MicroRNA miR-504 is known to be a p53-repressive miRNA, binds to the 3′ UTR of the protein p53 and promotes apoptosis and cell cycle arrest. In colon cancer cells, miR-504 represses the p53 protein level and reduces the p53-mediated apoptosis and cell-cycle arrest in response to stress [27, 38]. In gastric cancer, miR-504 down-regulation activates p53 through TFF1 [35]. MiR-203 restricts proliferative potential and induces cell-cycle exit in keratinocytes and promotes epidermal differentiation by repressing p63 [44]. miR-203 is down-regulated in HNSCC [38] and acts as a tumor suppressor in larynx carcinoma and may regulate expression of E-cadherin and CD44 suggesting a possible role in ECM [36]. miR-139 inhibits proliferation and metastasis of LSCC by targeting CXCR4 [37]. In gastric cancer cells, HER2 interacts with CD44 and up-regulates CXCR4 by inhibiting expression of miR-139 [45]. miR-133a is down-regulated in tongue cancer that is accompanied by overexpression of an oncogene, pyruvate kinase type M2 (PKM2) [46]. Further, we performed pathway analysis of the miRNA target genes using mirPath [47]. We found four different KEGG pathways, cell cycle, p53 signaling, viral carcinogenesis and pathways of cancer, to be significantly altered (with *P-*value 10^−13^, 10^−9^, 10^−7^ and 10^−6^ respectively). A list of all miRNA target genes, pathways, and gene annotations are provided in Supplementary Table 6. Previous studies showed that over-expression of miR-424 as a potential target for chemotherapy sensitization [48]. Hypoxia, a low oxygen condition, is an important process in carcinogenesis and known to play a role in the tumor microenvironment by allowing the development and maintenance of cancer cells [49]. MicroRNAs control gene expression and play an important role in cellular processes. The gene hypoxia inducible factor 1(HIF1) promotes survival of cells in low-oxygen conditions. The expression of miR-424 is stabilized by hypoxia through HIF1a and promotes angiogenesis[48, 50]. Although, the current data does not support the above hypothesis on the role of miR-424 in larynx and hypopharynx carcinoma, it is possible a similar mechanism is in operation in the tumor studied as well. It is shown that the micro RNA, miR-210, plays an important role in tumor invasion and proliferation, is induced by hypoxia and is associated with a poor prognosis in epithelial cancers [51-55]. Additionally, the protein VMP1 is identified as the direct and functional target of miR-210 in colorectal cancer [51]. Interestingly, we found a fusion event involving the gene *VMP1* in our study. It is early to state whether there is a relationship between the *CLTC-VMP1* fusion detected in our study and tumor invasion and proliferation. Taken together, it was interesting to see that some of the miRNAs identified in our study are related to regulation of cancer stem cell-like cells related markers affecting invasion and metastasis.

### Predicting larynx and pharynx carcinoma-specific minimal gene signature

In order to find a specific gene signature that distinguishes between the carcinoma of larynx and pharynx from those of the other subsites in the head and neck region, we performed random forest (RF) analysis [56] using gene expression data from this study and that from the TCGA dataset for validation. Random forest algorithm operates by constructing multiple decision trees based on a training set, and outputs the best prediction for the prediction set [56]. We also devised a method to calculate the overall score, an indication of strength of the signature. The score is calculated based on multiple factors including sensitivity (max possible = 100), number of iterations (max possible = 500), and number of genes in a set (min possible = 2; see Materials and Methods). Since HNSCC samples in the TCGA study belong to multiple different subsites of the head and neck region, we classified them into 2 types: those in larynx and/or hypopharynx sites (TCGA_L) and those in the oral cavity (TCGA_O). Each type was used independently with the samples in this study for training and predicting using the same matrix of samples and genes (Supplementary Table 7). The best prediction set contained 8 genes, ACPP, BRDT, DSC1, IFIT3, MAGEC2, MX1, TFF1 and WIF1 with a score of nearly 500 when we used the larynx and hypopharynx samples (including the larynx samples from TCGA) both as training and as prediction sets. Out of these genes, DSC1, TFF1 and WIF1 are known to be the markers of different carcinoma with altered expression levels [57-59]. When we performed random forest analysis using larynx, hypopharynx and oral cavity samples from TCGA as both training and prediction sets, we found a two gene signature, with genes BRDT and MAGEC2, come up with a score of 17200 indicating the possibility of specificity of these two genes in all three groups of cancer. MAGE proteins are a group of highly conserved eukaryotic proteins and about two thirds are aberrantly expressed in cancer tissues [60]. This data is supported by previous findings on altered expression of MAGEC2 and BRDT genes, in advanced head and neck cancer [61, 62]. Many of the MAGE proteins are linked to the process of p53-dependent apoptosis and help in tumor growth and metastasis [63]. Recently, there has been evidence that AMPK is inhibited by certain MAGE proteins (MAGE-A3 and MAGE-A6) in cell lines [64]. With additional work on these proteins in larynx and hypopharynx tumors, this could potentially be used to select patients to respond to AMPK-directed chemotherapy.

### Epigenetic silencing of WIF1

**Figure 3:**
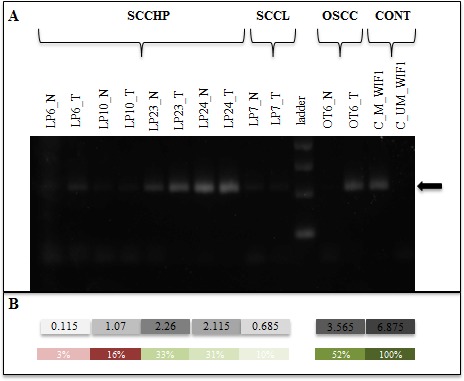
Promoter methylation study using Q-MSP for the gene WIF1 **A.** Products on an agarose gel after qMSP, SCCHP: squamous cell carcinoma of hypopharynx, SCCL: squamous cell carcinoma of larynx, OSCC: squamous cell carcinoma of oral cavity, N: matched normal, T: tumor, C_M: control DNA used with methylated primer, C_UM: control DNA used with unmethylated primer, arrow representing the amplified amplicon (210bp). **B.** Grey color intensity represents the extent of methylation (positive control being the highest), green: hyper-methylation, red: hypo-methylation, numbers inside the boxes are the ßßct values normalized against the internal beta-actin controls.

WIF1 is an extracellular *wnt* antagonist that functions as a tumor suppressor that is epigenetically silenced in various cancers and is involved in regulation of cancer stemness and senescence [26, 65, 66]. The epigenetic silencing of WIF1 was observed in lung, nasopharyngeal and esophageal tumors [58, 67]. The WIF1 gene was of particular interest to us since it is silenced in certain subsites of HNSCC [68] due to promoter hypermethylation. The WIF1 gene also was a part of the minimal gene signature from the random forest analysis. In order to understand the promoter hypermethylation of WIF1 and to understand whether such a mechanism is in operation in the larynx and hypopharynx carcinoma, we performed quantitative methylation-specific PCR (Q-MSP) for the WIF1 and calculated the extent of methylation in the samples. In the qMSP experiment, fifty percent of the hypopharynx samples had promoter hyper-methylation at WIF1 promoter and the rest had hypo-methylation at the same locus. In all samples, WIF1 gene was down-regulated but we could not find any correlation between WIF1 expression and its promoter methylation. For example, in the tumor sample LP6, the gene was down-regulated with log2FC of 5.3 but only 3% of the WIF1 promoters were methylated. The extent of down-regulation of WIF1 in the tumor LP7 was much smaller (Log_2_FC = 0.7) but WIF1 promoters were methylated to a larger extent (10%) than the LP6 tumor. In another tumor sample LP10, the log_2_FC for WIF1 was 2.7 while 16% of the WIF1 Promoters were hypo-methylated. As a control, we used a tumor from oral cavity (OT6) to study WIF1 promoter methylation and found very strong WIF1 hyper-methylation (52% methylation, Figure 3). In agreement with previous reports on HNSCC, we also found aberrant methylation of WIF1 but it was surprising that the expression of WIF1 was not strictly correlated with the extent of promoter hypermethylation in our samples unlike in nasopharynx[67]. The main predispositions for nasopharyngeal cancer are reported to be ancestry, Epstein-Barr virus (EBV) exposure, factors that result in very rare familial clusters and heavy alcohol intake[69-71]. This is different from those in larynx and hypopharynx cancer. Activation of *WNT* target gene may happen through multiple mechanisms; like presence of impaired scaffold proteins to bind beta-catenin that could result from the mutations in *APC* or *AXIN1* genes[72], mutations in the beta-catenin gene at its conserved sites [73], and epigenetic silencing of *WIF1* [74]. Although not frequent, a previous study showed mutation in *AXIN1* gene in 5% of oral squamous cell carcinoma tissue [72]. De-localization of beta-catenin is also reported in oral cancers previously[75]. Although we have not studied either the mutations in *AXIN1* gene or de-localization of beta-catenin in the larynx and hypopharynx samples, these can't be ruled out. Additionally, in all previous studies, where epigetic silencing of *WIF1* was shown to be involved in its down regulation, the frequency of hypermethylation varies anywhere between 35-92% depending on the subsite studied [68, 72]. In our study, we also found 40% of the tumors to have *WIF1* promoter hyper-methylation. Although the small sample size is a caveat in our observation, the results presented here suggest that the mechanism of *WIF1* silencing in hypopharynx and larynx tumors may not be linked to its promoter methylation. Expanding the study to include a larger sample size will shed more light on this.

## MATERIALS AND METHODS

### Informed consent and ethics approval

Informed consent was obtained voluntarily from each patient enrolled in the study. Ethics approval was obtained from the Institutional Ethics Committee of the Mazumdar Shaw Cancer Centre.

### Patient samples used in the study

Details of the tumor specimens and matched normal samples collected and used in the study are presented in Table 1. Only those patients with histologically confirmed squamous cell carcinoma that had at least 70% tumor cells in the specimen were recruited for the study. Patients included in this study underwent staging according to AJCC criteria at the Mazumdar Shaw Cancer Centre and the samples were accrued for the study. The treatment and surveillance were carried our as per NCCN guidelines (http://www.nccn.org/). Post-treatment surveillance was carried out by clinical and radiographic examinations as per the NCCN guidelines. In the discovery set, ten treatment-naïve (primary) and three recurrent patient samples were used for sequencing study. Out of the thirteen samples used in the discovery set, nine were tumors of the larynx and four of the hypopharynx. In the validation set of 18 tumors that included the discovery set, twelve samples were from larynx; three were from hypopharynx; and three other pharyngeal sites. Clinical details along with the treatment details for all samples are provided in Supplementary Table 1. All tissue samples were collected in RNAlater solution at the time of resection and stored at −80°C until further processing.

**Table 1:** Sample details used in the study. S: smoking, A: alcohol, C: chewing tobacco, N/A: not available

Sample name	Age	Gender	Subsite	Habits			TNM	Stage
				S	A	C		
LP1	70	M	Larynx	Y	Y	N	T3N0Mx	III
LP2	68	M	Larynx	Y	N	N	T4N2bMx	IVA
LP3	60	M	Larynx	N	N	N	T4N0Mx	IVA
LP4	62	M	Larynx	N	N	Y	T4N2cMx	IVA
LP5	64	M	Larynx	N	N	N	T1N0M0	I
LP6	57	F	Pharynx	N	N	N	T2N0M0	II
LP7	75	M	Larynx	N	N	Y	T4aN0Mx	IVA
LP8	60	M	Larynx	Y	N	Y	T3N0M0	III
LP9	40	F	Hypopharynx	N	N	Y	T3N0M0	III
LP10	54	M	Hypopharynx	Y	N	Y	T3N0Mx	III
LP11	46	M	Larynx	N	Y	N	T2N0M0	II
LP12	65	F	Larynx	N	N	N	T4aN2cM0	IVA
LP13	55	M	Hypopharynx	N/A	N/A	N/A	N/A	IVA

**Table 2:** Top over- and under-expressed genes in larynx and hypopharynx tumors

Gene ID	Log2FC	Gene ID	Log2FC	Gene ID	Log2 FC	Gene ID	Log2FC
CRNN	−7.36	ENDOU	−4.93	RELN	−3.8	FCER1A	−2.84
MUC21	−6.9	PADI1	−4.87	HOPX	−3.79	KRT34	−2.77
KRT4	−6.31	KRT13	−4.82	PPP1R3C	−3.75	TPRG1	−2.72
CYP4F29P	−5.77	MMRN1	−4.66	PKHD1L1	−3.63	ABI3BP	−2.68
SLURP1	−5.71	ATP6V0A4	−4.54	GBP6	−3.63	PRELP	−2.47
TMPRSS11B	−5.62	KRT78	−4.53	A2ML1	−3.36	ADH7	−2.44
TGM3	−5.48	SCEL	−4.32	CD24P4	−3.1	MMP11	2.37
CAPN14	−5.4	C2orf54	−4.22	IGSF10	−3.08	MMP12	3.6
SPINK8	−5.26	SH3BGRL2	−4.13	ANKRD35	−3.06	MMP10	4.7
MAL	−5.26	SPINK5	−4.06	SNORA20	−2.91	ZFPM2AS1	4.95
ANKRD20A11P	−5.24	LGI1	−3.98	SAMD5	−2.89	MMP1	5.41
ANGPTL1	−5.02	ANXA9	−3.84	SORBS2	−2.87		

### Library preparation and sequencing

Sequencing libraries were prepared for both whole transcriptome and small RNA libraries.

#### Whole transcriptome

Total RNA was isolated using Purelink RNA mini kit (Ambion, Life technologies) following manufacturer's instructions. Isolated total RNA was quantified using Qubit RNA Assay Kit (Invitrogen) before being used in the sequencing library preparation. For SOLiD whole transcriptome library preparation, two micrograms of total RNA was subjected to rRNA depletion using Ribominus Eukaryote Depletion System v2 (Ambion, Life technologies). Post rRNA depletion, the samples were quantified by Qubit RNA kit again and used for library preparation using SOLiD Total RNA seq kit, following the manufacturer's instructions. In brief, rRNA-depleted RNA was fragmented, cleaned, hybridized to adaptors, reverse transcribed, purified, size selected by AMPure beads over two rounds (to remove any fragments below 150bp), amplified, and finally, purified again. It was then quantified using Qubit HS kit (Invitrogen) and DNA 1000 Bioanalyzer (Agilent, 2100). Post library preparation, all the libraries were pooled in equimolar concentration and 0.6pM of the pooled library was used for the ePCR and enrichment step, as per the protocol. Following enrichment, the libraries were 3′ modified, loaded onto 4 lanes of the SOLiD flow cell and sequenced on SOLiD 5500xl sequencer. Data was obtained for 75×35 bp paired end reads (forward and reverse) in XSQ format.

#### Small RNA

Small RNA was isolated from tumor and matched normal samples using Ambion's MirVana kit following manufacturer's instructions. Small RNA library was prepared for 5 tumor-normal pairs and its quality was analyzed on RNA6000 nanochip using Agilent Bioanalyzer. Small RNA SOLiD sequencing libraries were prepared following manufacturer's instructions (Life Technologies). In brief, the library preparation involved hybridization and ligation, reverse transcription, purification of cDNA using Minelute PCR purification kit (Qiagen), gel size selection, amplification of the size selected cDNA and purification using Purelink PCR micro kit (Life technologies). For the gel size selection, the Novex TBE urea 10% - PAGE gel system (Life technologies) was used to excise bands between 60-80 nucleotides. Each gel piece was cut into 4 portions and two such portions were used for the process of amplification. Thus for each sample the cDNA library amplification was done in duplicates, with one gel piece in each tube. The final amplification of the size selected cDNA library was done for 15-18 cycles. Each of the libraries was labeled with a different barcode using SOLiD RNA Barcoding Kit, Module 1-16, which enabled multiplexing. The libraries were quantified using Qubit HS kit (Invitrogen) and the size distribution was analyzed using DNA HS chip on Agilent Bioanalyzer. Post library preparation, the five tumor/normal pairs (10 samples) were pooled together in equimolar concentration into a single library and 0.4pM of the pooled library was used for the ePCR, using e80 scale, and enrichment, as per manufacturer's instructions. Following enrichment, the library was 3′ modified, loaded onto a single lane of the SOLiD-6lane flowchip cell and sequenced on SOLiD 5500xl genetic analyzer system. Sequenced data was obtained (35 bp single-end reads) in the XSQ format and further used for downstream analysis using Lifescope.

### Read QC, alignment, read counts generation and gene-fusion detection

For all the tumor and normal samples, reads were filtered against tRNA, rRNA, adaptor and repeat sequences using Lifescope (v2.5-r2.5.1). The remaining reads were aligned to the hg19 reference sequence with default options. Of these, only primary alignments with minimum mapping quality of 10 were counted and the output was stored in a tab-delimited file, containing all gene annotations and their raw read counts. Additionally, putative gene-fusions in all samples were detected using the ‘splice finder’ module in Lifescope. A junction confidence value (JCV) ranging from 1 to 100 is specified with each predicted fusion event. The closer the JCV value to 100, the likelier the fusion event is to be real.

### Differential gene expression analysis

The gene-wise read counts of all the tumor and normal samples were pooled, and only those genes with a non-zero read count in at least one sample were selected. Normalization of the raw counts was done using the DESeq R package (R-3.1.0, DESeq-1.16.0) [76] and tested for differential expression with the following combinations: 1) all tumors vs their matched normal samples (referred as TN_P), 2) all tumor vs normal samples (matched & unmatched, TN_UP), and 3) pair-wise analyses for the individual tumor-normal pairs (T_P). From the DESeq output, all genes with a differential expression significance (padj) threshold of 0.05 (95% significance) in any of the three interpretations were selected as the genes of interest.

### Differential miRNA expression and pathway analyses

Pooled miRNA expression analysis was carried out using DESeq2 [77] R package (www.bioconductor.org). miRNA functional analyses using individual cancer-related pathway information were analyzed using the DIANA mirPath web server [47] that uses KEGG as the underlying database.

### Random Forest analysis

From the TCGA portal (https://tcga-data.nci.nih.gov/tcga/), raw RNA-seq read counts for matched tumor-normal TCGA HNSCC samples were downloaded and normalized using DESeq. For each sample pair, gene-wise tumor vs normal ratios of normalized counts, called fold changes (FC), were calculated. Genes with fold change calculations of less than 1 (FC<1) were taken as down-regulation (D), genes with FC>1 were taken as up-regulation (U) and FC=1 meant no change (X) in the expression of the gene in tumor with respect to matched normal. Furthermore, based on the subsite, all the above TCGA samples were classified into 2 types - TCGA_L (larynx subsite, *N*=11 pairs), TCGA_O (all subsites in the oral cavity, *N*=29 pairs). Finally, for the set of significantly differentially expressed genes from SCCL and SCCHP samples, FC values (in D, U or X notations) and TCGA_L or TCGA_O samples were used as input to the machine learning algorithm random forests [56]. We ran the computation with different seeds (1-500) that controlled for the randomness in the experiment and calculated a RF score (*R*) by using a formula:

$$R=\frac{S(ni)}{\left(ng\right)}$$

where S is the sensitivity of detection, ni is the number of iterations in which a predictive set of genes is repeated in a set of 500 and ng is the number of genes in the predictive set. The highest score achievable using this method is 25000.

### Validation of genes with significant expression change

Genes found to be significantly up- and down-regulated in larynx and hypopharynx tumors were validated using quantitative PCR (qPCR) experiments in eighteen tumor samples (validation set in Supplementary Table 1). Total RNA was isolated from normal and tumor samples using RNeasy Mini Kits (Qiagen). Total RNA (125 ng) was reverse transcribed using SuperScript III First-Strand Synthesis kit (Invitrogen), following manufacturer's instructions. The qPCR primers for 14 most significantly altered genes were designed using NCBI Primer-BLAST. Details of the primer sequence, their annealing temperature, amplicon sizes are provided in Supplementary Table 2. The reaction mix was denatured at 95°C for 3 minutes and amplified for 40 cycles at 95°Cfor 3 seconds with an annealing temperature depending on the primer used for 30 seconds. The amplification was followed by dissociation curve analysis. *Beta*-actin housekeeping gene was used to normalize qPCR data using the Kapa SYBR Fast qPCR Kits (Kapa Biosystems).

### Quantitative Methylation-Specific PCR (qMSP)

Genomic DNA was isolated from normal and tumor samples using DNeasy Blood & Tissue Kit (Qiagen), following the manufacturer's instructions. Genomic DNA (500 ng) was bisulfite converted using EZ DNA Methylation Kit (Zymo Research), and amplified using two primer sets, one set specific for methylated and the other for non-methylated DNA. Methylation-specific primers were designed using MethPrimer tool (http://www.urogene.org/methprimer/). Sequences of the methylation-specific primers, annealing temperature and amplicon size is provided in Supplementary Table 2. Quantitative-MSP reactions were carried out using Kapa SYBR Fast Kits (Kapa Biosystems). The reaction mix was denatured at 95°C for 3 minutes and amplified for 40 cycles at 95°C3 seconds, annealing temperature depending on the primer for 30 seconds, followed by extension at 72°C for 1 minute. The amplification was followed by dissociation curve analysis. Universal Methylated/Un-methylated Human DNA Standards (Zymo Research) were used as positive and negative assay controls for qMSP. Quantitative methylation in each sample was normalized using the methylated and un-methylated primers for beta-actin. Post-qMSP, the products were loaded on a gel for visualizing the methylation difference in WIF1 promoter.

The scripts used to analyze and interpret data are provided in Supplementary Text1.

## SUPPLEMENTARY TABLES


